# Genetic Influence on LDL-Cholesterol Levels: Role of Polygenic Risk Scores and Lp(a) Beyond Monogenic Hypercholesterolemia

**DOI:** 10.3390/genes17060721

**Published:** 2026-06-21

**Authors:** Martina Ferrandino, Ylenia Cerrato, Gabriella Iannuzzo, Ilenia Lorenza Calcaterra, Matteo Nicola Dario Di Minno, Giuliana Fortunato, Maria Donata Di Taranto

**Affiliations:** 1 Dipartimento di Medicina Molecolare e Biotecnologie Mediche, Università Degli Studi di Napoli Federico II, Via Pansini 5, 80131 Naples, Italy; martina.ferrandino@unina.it (M.F.); ylenia.cerrato@unina.it (Y.C.); fortunat@unina.it (G.F.); 2 CEINGE Biotecnologie Avanzate Franco Salvatore, Via Gaetano Salvatore 486, 80145 Naples, Italy; 3 Dipartimento di Medicina Clinica e Chirurgia, Università Degli Studi di Napoli Federico II, Via Pansini 5, 80131 Naples, Italy; gabriella.iannuzzo@unina.it (G.I.); ilenialorenza.calcaterra@unina.it (I.L.C.); matteo.diminno@unina.it (M.N.D.D.M.)

**Keywords:** hypercholesterolemia, genetics, familial hypercholesterolemia, polygenic risk score, lipoprotein(a), LDL-cholesterol, single-nucleotide polymorphism, molecular diagnosis

## Abstract

High levels of low-density lipoprotein cholesterol (LDL-c) have been recognized as the main causal factor of atherosclerotic cardiovascular disease (ASCVD) and are influenced by both genetic and environmental factors. Among genetic determinants, Familial Hypercholesterolemia (FH) is the most common monogenic disorder, caused by rare high-impact variants in genes involved in LDL uptake. Other monogenic causes of hypercholesterolemia include sitosterolemia, cerebrotendinous xanthomatosis and lysosomal acid lipase deficiency (LALD). However, monogenic disorders only account for a small proportion of inherited hypercholesterolemia. In many individuals, increased LDL-c levels are caused by the contemporary presence of different single-nucleotide polymorphisms (SNPs) with a moderate/low impact. These SNPs could be summarized through polygenic risk scores (PRS) that attribute relative weight to each of these. Another genetic determinant of hypercholesterolemic phenotypes is high levels of lipoprotein(a)—Lp(a). Lp(a) is an LDL particle modified by the binding of apolipoprotein(a)—apo(a)—which represents an independent risk factor for ASCVD. Lp(a) levels are mainly genetically determined by variation in the number of kringle IV type 2 (K-IV_2_) repeats, as well as by several SNPs, and remain stable throughout life. The aim of this narrative review is to report an updated overview of the genetic mechanisms underlying hypercholesterolemia, including monogenic disorders, PRS and Lp(a), focusing on their potential repercussion in clinical practice by the integration into cardiovascular risk stratification beyond traditional clinical assessment. This integration could lead to a more comprehensive and individualized approach to cardiovascular prevention, with emerging perspectives including the possible use of artificial intelligence (AI).

## 1. Introduction

**Figure 1:**
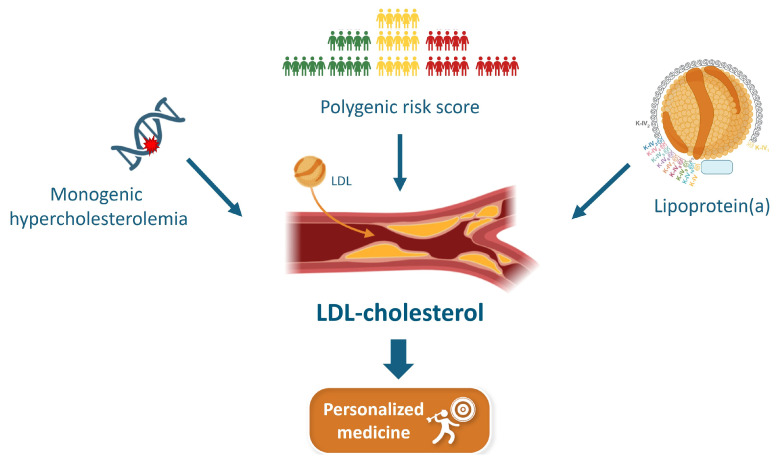
Genetic determinants of LDL-cholesterol. High levels of LDL-cholesterol may result from rare pathogenic variants causing monogenic hypercholesterolemia, from the cumulative effect of different single-nucleotide polymorphisms summarized by polygenic risk scores and from elevated lipoprotein(a)—Lp(a)—levels, which are mainly genetically determined.

Atherosclerotic cardiovascular disease (ASCVD) is the main cause of mortality worldwide, with low-density lipoprotein cholesterol (LDL-c) as the main driver of its pathogenesis [1]. Atherosclerosis is caused by the accumulation of LDL-c and other apolipoprotein B (ApoB)-containing lipoproteins into the arterial wall, with increasing damage depending on both the magnitude and the duration of the exposure. So, maintaining low levels of LDL-c over time, following the emerging scientific consensus of ‘lower is better, and earlier is better’, could lead to a substantial reduction in ASCVD risk [2]. The aim of this narrative review is to provide an updated overview of the complex genetic architecture underlying hypercholesterolemia and increased cardiovascular risk by discussing the contribution of monogenic disorders, polygenic risk scores (PRS) and lipoprotein(a)—Lp(a)—to increased LDL-c levels (Figure 1).

With respect to other reviews focused mainly on the genetics of Familial Hypercholesterolemia (FH) (some recent and relevant ones are [3,4,5,6]), the novelty of this review lies in an updated overview of the current scientific evidence on a broader spectrum of genetic determinants of elevated LDL-c levels, highlighting the importance of considering different genetic scenarios in both the diagnostic workup and cardiovascular risk assessment. It also discusses some methodological aspects and contemporary management strategies in light of the latest therapeutic advances tailored to the underlying alteration. New perspectives on personalized medicine are also discussed, focusing on possible integration strategies using artificial intelligence (AI). This could lead to improved cardiovascular prevention with a more comprehensive and individualized approach and, consequently, better patient management.

A systematic literature search strategy and study selection process were not applied; however, we conducted a selective literature search of PubMed, Scopus and Web of Science, using combinations of keywords such as “hypercholesterolemia”, “genetics”, “familial hypercholesterolemia”, “LDL-cholesterol”, “atherosclerosis”, “atherosclerotic cardiovascular disease”, “genetic diagnosis”, “heterozygous familial hypercholesterolemia”, “homozygous familial hypercholesterolemia”, “oligogenic familial hypercholesterolemia”, “autosomal recessive hypercholesterolemia”, “sitosterolemia”, “cerebrotendinous xanthomatosis”, “lysosomal acid lipase deficiency”, “lipid lowering therapies”, “polygenic risk scores”, “genetic risk scores”, “single nucleotide polymorphism”, “LDL-cholesterol polygenic risk scores”, “coronary artery disease polygenic risk scores”, “cardiovascular risk stratification”, “cardiovascular risk prevention”, “genome association studies”, “lipoprotein(a)”, “*LPA* gene”, “lipoprotein(a) measurement”, “lipoprotein(a)-lowering therapies”, “artificial intelligence”, and including meta-analyses, original articles, clinical guidelines and reviews. Bibliographical references from the retrieved papers were also considered to find relevant studies that might have been missed during the initial search.

## 2. Monogenic Hypercholesterolemia

Monogenic hypercholesterolemia includes different genetic disorders characterized by high LDL-c levels caused by rare pathogenic variants in one of the genes involved in lipid metabolism (Table 1). Among monogenic disorders, the most frequent is FH.

### 2.1. Familial Hypercholesterolemia (FH)

FH is a frequent genetic disease with an autosomal semi-dominant transmission, characterized by high levels of LDL-c that could lead to premature atherosclerosis and increased risk of coronary artery disease (CAD) [7]. It is mainly caused by rare pathogenic variants in three genes encoding proteins involved in LDL uptake [8]. Most FH-causative variants are loss-of-function variants in the *LDLR* gene, encoding for LDL receptor (LDLR). Loss-of-function variants in the apolipoprotein B (*APOB*) gene and gain-of-function variants in proprotein convertase subtilisin/kexin type 9 (*PCSK9)* gene are less frequent [9].

There are two forms of disease: the heterozygous and the homozygous forms. The most frequent is heterozygous FH (HeFH), characterized by the presence of a single variant on one of the two alleles of the FH-causative genes and with an estimated prevalence of 1:313 [10]. This prevalence has also been confirmed by a large-scale analysis of whole-genome sequencing data from the 100,000 Genomes Project [11].

The homozygous form (HoFH) is caused by the presence of two pathogenic variants. It could be caused by the same variant present on the two alleles of the same gene (true homozygosis or biallelic identical variant), by two different variants on the two alleles of the same gene (compound heterozygosis or biallelic different variants) or by two heterozygous variants in two different causative genes (double heterozygosis or digenic variants) [12,13]. It is characterized by a more severe phenotype with very high LDL-c levels and an early onset of ASCVD [13].

The HoFH is a rarer condition with an estimated prevalence approximatively of 1:300,000, with a higher frequency in populations with a founder effect [12,13]. Although only a few epidemiological data are available, in Southern Italy we reported an estimated prevalence of 1:320,000, with an increase to 1:286,000 thanks to the efforts in the identification of HoFH patients made in recent years. However, HoFH is still undiagnosed, so the frequency could be even higher [14,15].

#### 2.1.1. Genetic Diagnosis

In patients with clinical suspicion of FH, genetic testing is important to confirm the presence of pathogenic variants and to estimate inheritance risk, allowing cascade screening [16]. In fact, it has been demonstrated that, at any LDL-c levels, patients carrying an FH-causative variant have a higher risk of CAD than those without FH-causative variants [17]. Also, differences in genetic status could be associated with differences in plasma lipidomic profiles, particularly in sphingomyelin levels, supporting their emerging role in hypercholesterolemia and cardiovascular disease (CVD) [18,19].

Genetic analysis is currently made by Next Generation Sequencing (NGS), which, by analyzing all FH-causative genes at the same time, allows the identification of many variants. However, the main issue remains the correct interpretation of their pathogenicity. In fact, a lot of variants are classified as uncertain significance variants (USVs) because there is not enough evidence supporting their pathogenic or benign effect [20]. Functional studies could allow us to overcome this problem of uncertain classification by providing strong evidence of pathogenicity/benignity [21,22,23]. An approach has recently been proposed aiming at functionally characterizing all possible *LDLR* coding variants [24]. Conversely, functional characterization of *APOB* and *PCSK9* variants is more complex, requiring several experimental approaches [25,26] and thus remains mostly undone.

No FH-causative variant is detected in a substantial proportion of patients with clinical suspicion of FH, so it remains widely underdiagnosed and undertreated, in particular in children and young people [6,27,28]. Globally, only 2% of patients receive a genetic diagnosis before the age of 18 years. Considering the cumulative exposure to high LDL-c levels, genetic diagnosis occurs too late, with a mean age of 43 years in men and 46 years in women [29].

Universal lipid screening in children aged 9 to 11 years has been recommended and may significantly improve the identification of pediatric FH patients, allowing the early beginning of therapy [30,31,32]. This is particularly relevant considering that increased carotid intima-media thickness (c-IMT) has been observed in untreated HeFH children compared with their unaffected siblings, even before the age of 8 years [33].

#### 2.1.2. Therapies for FH

Obtaining a molecular diagnosis is also important to define the best therapeutic options for the patient [34]. In fact, it has been observed that genetic status could influence clinical outcomes of PCSK9 inhibitors (PCSK9i), in terms of both LDL-c levels and carotid stiffness [35,36]. Different PCSK9i have been developed, both monoclonal antibodies (evolocumab and alirocumab) that act by blocking the interaction of circulating PCSK9 and LDLR, and short interfering RNA (siRNA) (inclisiran) which prevents the intracellular synthesis of PCSK9 [37].

In HoFH patients, traditional lipid lowering therapies (LLTs) alone do not allow to reach LDL-c target levels, making plasma apheresis an essential treatment [38,39]. In recent years, novel therapies independent of LDLR activity have been developed [40].

Among these, lomitapide act by the inhibition the microsomal triglyceride transfer protein (MTTP), leading to a reduction of above 60% in LDL-c levels without significant impact on liver status [41]. A great variability among patients has been observed [42,43] and could be partially explained by sex differences [44], even if HoFH affects men and women equally [45].

Evinacumab is a monoclonal antibody directed against Angiopoietin-like protein 3 (ANGPTL3) that allows a mean reduction in LDL-c levels of about 50%, independently of LDLR activity [46,47]. This therapy was recently approved for use in children, representing a valuable tool for CVD prevention in this severe form of hypercholesterolemia.

### 2.2. Additional FH Causative Genes and Oligogenic FH

Beyond the three classical FH-causative genes involved in FH, an important contribution to the disease could be due to other genes involved in lipid metabolism, such as the apolipoprotein E (*APOE*) gene and the low-density lipoprotein receptor adaptor protein 1 (*LDLRAP1*) gene.

The role of *APOE* variants in the alteration of lipoprotein metabolism is well known [48]. It has been demonstrated that ApoE is important for lipoprotein clearance, representing the main ligand of chylomicrons and very low-density lipoproteins (VLDL) [49]. Only one pathogenic variant (p.Leu167del) has been described in the *APOE* gene as associated with FH. This variant potentially compromises ApoE structure, affecting its capacity to bind lipids and its affinity to LDLR, causing increased LDL-c levels [50,51].

Pathogenic variants in both alleles of the *LDLRAP1* gene are causative of autosomal recessive hypercholesterolemia (ARH). LDLRAP1 protein is important for LDLR interaction with clathrin-coated pits, so the absence of this protein enables the internalization of the LDL-LDLR complex [52]. Several cases have been described worldwide; however, ARH remains a very rare form of monogenic hypercholesterolemia with a cardiovascular prognosis like that of HoFH [53]. One functional copy of the *LDLRAP1* gene seems to be enough to maintain its functionality; in fact, heterozygous *LDLRAP1* variants have been associated with normal LDL-c levels [54]. However, there have been reported cases of heterozygous carriers of *LDLRAP1* variants with abnormal lipid profiles and premature cardiovascular events [55,56].

Further studies are needed to better understand the role of this gene in hypercholesterolemia. In the meantime, its inclusion in the genetic analysis of patients with clinical suspicion of FH is important for the identification of new cases.

Another genetic condition characterized by increased LDL-c levels is oligogenic FH, caused by the contemporary presence of a pathogenic variant in one of the classical FH-causative genes and in one of the other cholesterol-impacting genes (*ABCG5*, *ABCG8*, *APOE*, and *LDLRAP1*). It has been found that patients with oligogenic FH have higher LDL-c levels than patients with “classical FH”, so the presence of variants in the accessory genes could contribute to the worsening of the phenotype [57].

### 2.3. Other Monogenic Hypercholesterolemia

Other monogenic diseases, such as sitosterolemia, cerebrotendinous xanthomatosis (CTX) and lysosomal acid lipase deficiency (LALD), could lead to increased LDL-c and total cholesterol levels [6].

Sitosterolemia is an autosomal recessive disease characterized by severe xanthomatosis and increased levels of plasma plant sterols and cholesterol. Increased levels of plasma plant sterols, in particular sitosterol and campesterol, are due to intestinal hyperabsorption and low bile excretion. In fact, generally, plasma plant sterols are poorly adsorbed in healthy individuals (about 5%) [58]. It is caused by biallelic pathogenic variants in the *ABCG5* and *ABCG8* genes. These genes form a heterodimer involved in the transmembrane transport of sterols and located on the apical membrane of enterocytes and hepatocytes [59]. A diagnosis of sitosterolemia was made in 2.7% of Brazilian FH-negative patients and it was also demonstrated that heterozygous *ABCG5* or *ABCG8* variants were 8.3× more frequent in this cohort than in the general population. This suggests that also heterozygous pathogenic variants in *ABCG5/ABCG8* could have a role in hypercholesterolemia [60].

CTX is an autosomal recessive disease characterized by extensive xanthomatosis that mimics that of FH. Increased levels of LDL-c were also observed, although variable manifestations could be possible, with moderately higher or even normal levels. It is caused by biallelic pathogenic variants in the *CYP27A1* gene, encoding the mitochondrial cytochrome P450 sterol 27-hydroxylase enzyme. The loss of function of this enzyme causes errors in bile acid synthesis and lipid accumulation [61]. Biallelic pathogenic variants in *CYP27A1* have been identified both in patients clinically misdiagnosed as FH and in HeFH patients, worsening their phenotype with a more severe progression of xanthomatosis and atherosclerosis [62,63]. There is also evidence of heterozygous pathogenic variants in *CYP27A1* identified in FH-negative patients [64].

LALD is a rare multi-systemic disease with autosomal recessive inheritance, caused by pathogenic variants in the *LIPA* gene, encoding the lysosomal acid lipase enzyme, essential for the degradation of cholesteryl esters. It is characterized by high levels of LDL-c, total cholesterol and triglycerides and low levels of high-density lipoprotein cholesterol (HDL-c), associated with elevation of the hepatic transaminases, aspartate aminotransferase (AST) and alanine aminotransferase (ALT) [65]. In the Portuguese FH Study, four children with LALD were identified among patients misdiagnosed with FH [66].

#### Therapies for Rare Monogenic Hypercholesterolemia

Differential diagnosis is important to better treat these patients; in fact, each of these diseases have a different approach of personalized medicine.

In sitosterolemia the aim of the therapy is to reduce both the plasma sterols and the cholesterol levels. So, the first-line treatment is dietary restriction of plant sterols [67]. In addition to diet, bile acid sequestrants and ezetimibe can lead to an additional reduction in plasma sterol levels. Ezetimibe, through the binding of the Niemann–Pick C1-like 1 (NPC1L1) cholesterol transporter, blocks the absorption of both cholesterol and plant sterols [68]. CTX is usually treated with supplementation of chenodeoxycholic acid that inhibits bile acid synthesis, decreasing cholestanol production and accumulation [62]. For LALD, the better treatment seems to be enzyme replacement therapy (ERT), in which patients receive a recombinant enzyme agent biweekly [65]. In some cases, a combination therapy of ERT and hematopoietic stem cell transplant (HSCT) could be considered [69]. It has been reported that, in the long term, HSCT could lead to the progressive replacement of hematopoietic lineage cells with donor-derived enzyme competent cells, allowing to stop ERT [70].

## 3. Polygenic Risk Scores

Among patients with inherited hypercholesterolemia, monogenic causes account for only a small percentage, suggesting that increased LDL-c levels are usually caused by more complex genetic mechanisms. In these hypercholesterolemic patients without rare pathogenic variants causing monogenic hypercholesterolemia, increased LDL-c levels may result from the cumulative effect of different single-nucleotide polymorphisms (SNPs), with small individual impact on the phenotype.

Over the past decade, there has been increasing interest in PRS thanks to the advancement of genome-based approaches, such as NGS and genome-wide association studies (GWAS), which have enabled the identification of many common variants with a moderate/low impact on different phenotypes, such as increased LDL-c levels and CAD. Based on the concept of the cumulative effect of several SNPs, different PRS have been developed, usually constructed as weighted scores considering the effect size of each SNP, determined by GWAS, and multiplying it for the number of risk alleles [71]. The performance of each of these PRS may vary across populations because of the genetic differences among ancestries [72].

### 3.1. PRS and LDL-c Levels

Several weighted PRS for LDL-c levels (LDL-c PRS) have been developed to capture the cumulative genetic contribution to increased LDL-c levels. One of the first and most studied has been developed in the UK population and built on 12 SNPs [73] and then refined to reduce the number to the 6 most impacting SNPs [74]. Since then, different LDL-c PRS including a great number of SNPs associated with LDL-c levels have been developed (Table 2), reaching more than 2 million SNPs (2M-SNP) [75].

A recent meta-analysis of different GWAS developed and compared 4 LDL-c PRS based on 165, 284, 638 and 1633 SNPs, demonstrating that these scores performed equally in LDL-c levels prediction and have a similar ability to discriminate between hypercholesterolemic patients and healthy controls [76]. Among these, considering comparable performance, the 165 SNPs score, requiring a small panel, could be more compatible with clinical settings. It has also been shown that this score performed better than the 12 SNPs one, allowing to identify a genetic cause in a greater percentage of hypercholesterolemic patients [76].

Different research studies about LDL-c PRS revealed the association of high score values with hypercholesterolemia, but do not provide a clear cut-off value that could be used as a diagnostic criterion to identify patients with polygenic hypercholesterolemia (Table 2). In addition, even when cut-off values have been proposed, several false-positive and false-negative subjects were observed, impacting the clinical utility of LDL-c PRS.

The identification of a genetic predisposition could be performed since birth, making the patients more conscious of their risk and more compliant with a healthy lifestyle and eventual therapies [77].

Beyond their role as genetic determinants of increased LDL-c levels, LDL-c PRS could also modify the phenotypic expression of FH. In fact, it was observed that patients with pathogenic variants and high values of 12 SNPs and 6 SNPs scores have higher levels of LDL-c than patients with lower values of both scores, representing a possible cause of phenotype variability of FH patients. This was observed with a greater impact in children than in adults, suggesting that the genetic predisposition of increased LDL-c levels must be investigated since childhood [78]. It has also been observed that low LDL-c PRS could lead to incomplete penetrance of FH [79]. Overall, these findings highlight that LDL-c PRS could be both a cause of hypercholesterolemia, an alternative to monogenic diseases, and a modifier factor, contributing to the phenotypic variability of FH.

Furthermore, LDL-c PRS above the 95th percentile have been associated with increased CVD risk in both monogenic and polygenic hypercholesterolemia [80,81]. So, the clinical utility of these scores is not limited to a genetic explanation of hypercholesterolemia but could also improve CVD risk stratification.

**Table 2:** LDL-c PRS associated with increased LDL-c levels.

Number of SNPs	Score Model	Score Derivation	Sample Size	Cut-Off Values	References
12	Weighted	GWAS of Europeans [82]	640 UK HC patients; 3020 controls; validation on 727 Belgian HC patients	≥10th percentile	Talmud et al. 2013 [73]
6	Weighted	GWAS of Europeans [73,82]	1158 HC patients (638 Dutch adults, 22 Dutch children, 128 Greek children, 76 Canadian adults, 202 Italian adults, 29 Polish adults and 63 Israeli adults); 3020 controls	≥2th quartile	Futema et al. 2015 [74]
4	Weighted	GWAS of East Asians [83]	97 Korean HC patients; 2274 controls	n.s.	Kwon et al. 2015 [84]
10	Weighted and unweighted	GWAS of several ancestries [82,85,86]	313 Ontario HC patients; 1092 controls from the phase 1 of the 1KG project	≥90th percentile	Wang et al. 2016 [87]
2M	Weighted	WGS of several ancestries	16,324 individuals (4064 from FHS; 1083 from OOA; 3247 from JHS; 4510 from MESA; 1165 from FIN and 2255 from EST)	≥5th percentile	Natarajan et al. 2018 [75]
28	Weighted	GWAS of several ancestries [75,85,86]	1120 HC patients (262 from the BCFH study; 552 from CNMA study and 306 from UK Biobank cohorts)	≥80th percentile	Trinder et al. 2020 [79]
165	Weighted	Meta-analysis [88] of the 3 most recent GWAS [82,86,89] from the GLGC	785 French HC patients; 1938 controls (1082 from the MONA LISA study and 856 from the FGR consortium)	≥75th percentile	Vanhoye et al. 2023 [76]
284	Weighted				
638	Weighted				
1618	Weighted				

SNPs: Single-nucleotide polymorphisms; GWAS: genome wide association study; HC: hypercholesterolemic; n.s.: not specified; 1 KG: 1000 genomes; WGS: whole-genome sequencing; FHS: Framingham Heart Study; OOA: Old Order Amish; JHS: Jackson Heart Study, MESA: Multi-Ethnic Study of Atherosclerosis; FIN: FINRISK Study; EST: Estonian Biobank; BCFH: British Columbia FH; CNMA: Nutrition, Metabolism and Atherosclerosis Clinic; GLGC: Global Lipids Genetics Consortium; MONA LISA: Monitoring National du Risque Arteriel, National Monitoring of Arterial Risk; FGR: FranceGenRef.

### 3.2. PRS and Cardiovascular Risk

In contrast to LDL-c PRS, which capture genetic determinants of increased LDL-c levels, CAD PRS are designed to estimate CVD risk by integrating different biological pathways beyond lipid metabolism. Accordingly, although some SNPs overlap with those of the LDL-c PRS due to shared cholesterol-related pathways, they represent only a minority of CAD-associated SNPs, as CAD PRS also capture additional pathways dysregulated in CAD such as inflammation, cellular proliferation, and vascular tone [90,91]. Since the advent of GWAS, several CAD PRS have been developed. Detailed discussions of currently available CAD PRS and their strengths and limitations are available elsewhere [91,92,93,94]. Among the other, a CAD PRS comprising about 1.7 million SNPs has been demonstrated to have a comparable or even better predictive capacity than the conventional risk factors, representing a test that may be applied before the manifestation of clinical risk factors [95]. CAD PRS are associated with increased CVD risk with an effect size like that of LDL-c levels. Furthermore, it has recently been showed that patients with a moderate increase in LDL-c levels, but high CAD PRS, have a CVD risk comparable to that of patients with very high LDL-c levels, supporting the use of LDL-c threshold PRS-adjusted in primary prevention [96].

A recent study, using a CAD PRS of 6.6 million SNPs, allowed the identification patients with an increased CVD risk, comparable to that of HeFH [97]. The correlation between different CAD PRS and CVD risk in FH patients has been investigated in different studies [3]. One of the most recent shows showed that FH patients with a high CAD PRS (>75th percentile) had a twofold increased risk of CAD compared with those with lower CAD PRS values (≤75th percentile), independently of other clinical factors. Furthermore, a very low CAD PRS (<5th percentile) was associated with significant protection against cardiovascular events, although it should never be considered as conferring complete protection [98]. Overall, these findings suggest that CAD PRS could also act as CVD risk modifiers, leading to an increased CVD risk in FH patients [99].

CAD PRS could be useful not only in primary prevention but also in secondary prevention to better stratify patients that already experienced myocardial infarction (MI). It has been observed by different post hoc analysis that, among patients with well-known ASCVD, those with PRS >90th centile have a greater risk of other CV events [91,100].

Given their stability throughout life, not being influenced by age or other biological factors, CAD PRS determination since birth could be useful for early risk stratification. These findings highlight the importance of the early genetic characterization of patients without ASCVD, improving their therapeutic management [91].

### 3.3. Integration of CAD PRS with Clinical Score

Growing evidence supports the importance of integrating CAD PRS with clinical risk scores, such as the SCORE2/SCORE2-OP and PREVENT equation, to improve CVD risk prevention [16,101]. In particular, CAD PRS have been shown to improve CVD risk reclassification compared with clinical risk scores alone across different studies [102,103,104]. Relative genetic risk and absolute clinical risks appear to be independently associated with total CVD risk, supporting the utility of their integration in a combined risk prediction model. A multiplicative model has been proposed and validated in a large cohort, resulting in an improved risk stratification with a remarkable reclassification of many patients [104].

This integrated approach seems to be particularly useful in patients classified at intermediate risk according to SCORE2/SCORE2-OP. In this group, CAD PRS could allow the early identification of patients at higher risk who could benefit most from emerging therapies. Notably, patients reclassified into higher-risk categories using this integrated model experienced CVD events about twice as often as expected based on clinical risk score alone, further supporting the clinical relevance of this combined approach [104].

This could be particularly relevant in younger individuals, in whom the CVD risk is often underestimated [105].

### 3.4. Limitations of PRS

Despite their promising clinical applications, several limitations, mainly related to a lack of standardization, currently restrict the implementation of both LDL-c and CAD PRS in clinical practice. These include differences in score construction, reporting formats (i.e., raw values or percentiles), selection of included SNPs, and cut-offs used to define high PRS values [71].

In addition, most GWAS have been conducted in European populations, resulting in reduced performance of PRS in non-European individuals [72]. Only a few data are available from GWAS and LDL-c PRS validation studies conducted in non-European individuals, such as East Asian cohorts, including Korean populations [83,84]. This limitation may substantially affect the external validity of LDL-c PRS and their applicability in global clinical practice, as risk prediction accuracy may vary across different ancestry groups. Consequently, the clinical implementation of LDL-c PRS in underrepresented populations may exacerbate existing health inequities in CVD risk assessment and prevention. However, even among European individuals some bias could be due to the use of different training and validation cohorts, further limiting PRS generalizability [106].

Another important limitation affecting the clinical implementation of LDL-c PRS is the lack of universally accepted cut-off values and standardized interpretation criteria, potentially leading to inappropriate risk estimation. Due to these aspects, the diagnostic utility of LDL-c PRS in routine clinical practice remains limited.

The same limitations are present for CAD PRS, as remarked by the recent American guidelines on CVD prevention, which only recently recognized them as CVD risk modifiers [16]. In fact, also for CAD PRS, specific cut-off values are not usually indicated and studies on ancestry remain limited, reducing their diagnostic utility [71]. These aspects highlight the need to develop standardized approaches and additional studies on ancestry to obtain adjusted analyses [107,108].

## 4. Lp(a)

In addition to polygenic contribution to LDL-c levels variability, other independent genetic factors, such as Lp(a), could play an important role in determining inherited hypercholesterolemia. Recent studies reported higher Lp(a) levels in variant-negative FH patients, both adults and children, highlighting that high Lp(a) levels could underlie hypercholesterolemic phenotype [109,110]. Lp(a) is an LDL-like lipoprotein containing one molecule of apolipoprotein(a)—apo(a)—covalently bound to apolipoprotein B (ApoB). Although considered an independent risk enhancer [111,112,113], Lp(a) levels are not yet included in the main scores (SCORE2/SCORE2-OP and PREVENT equation) for estimation of CVD risk [7,16,101]. Its introduction could be useful considering that there are several studies reporting on the additional and improving role of Lp(a) in CVD risk prediction allowing the identification of patients that could benefit from more intensive LLT [114,115].

### 4.1. Epidemiology

Lp(a) levels show great interindividual variability, ranging from <0.2–750 nmol/L (<0.1 mg/dL to >300 mg/dL). This elevated variability is mainly due to genetic factors with a minimal influence of lifestyle [116].

In the UK Biobank, it was observed that median Lp(a) levels increase going from Chinese to White, South Asian and Black individuals. In populations with low levels, the distribution is skewed with a tail toward high concentrations, while in populations with higher levels the distribution appears to be less skewed than in other ethnic groups. This highlights the importance of ethnicity’s influence on Lp(a) levels. These ethnic differences are relevant not only for Lp(a) levels distribution, but may also influence CVD risk estimation, as universally accepted cut-off values could not be considered. However, despite this difference, high levels of Lp(a) appear to be associated with CVD risk in all ethnic groups [111,114,117]. Also, sex differences have been observed; in fact, it has been observed that Lp(a) levels are higher in women than in men, in particular in menopausal age [112].

### 4.2. Structure and Genetics

The main characteristic that allows us to distinguish Lp(a) from LDL particles is the presence of the apo(a), which is covalently linked to ApoB by a disulfide bridge.

Apo(a) is a plasminogen-like glycoprotein encoded by the *LPA* gene, which originated from a translocation and duplication of a portion of the plasminogen (*PLG*) gene. Compared with plasminogen, apo(a) lacks kringle domains I–III and exhibits an expansion of the kringle IV domain, differentiated in ten different subtypes. Among these, kringle IV type 2 (K-IV_2_) is characterized by a variable number of repeats, with up to more than 40 copies [112,118].

**Figure 2:**
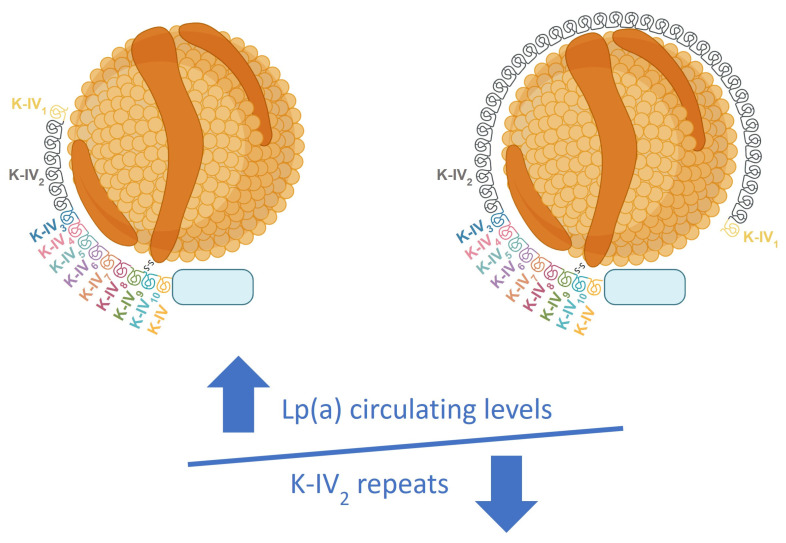
Inverse relationship between the number of kringle IV type 2 (K-IV_2_) repeats and Lipoprotein(a)—Lp(a)-levels. There are different isoform sizes of apolipoprotein(a)—apo(a)—covalently linked to apolipoprotein B to form Lp(a), due to the different number of repeats of the K-IV_2_ domain. Smaller apo(a) isoforms are usually associated with higher Lp(a) levels, although this relationship is modulated by several single-nucleotide polymorphisms.

The primary genetic determinant of Lp(a) levels is the number of K-IV_2_ repeats, explaining ∼30–70% of the variability. There is an inverse relationship between the number of K-IV_2_ repeats and Lp(a) levels; in fact, individuals with a low number of K-IV_2_ repeats have smaller apo(a) isoforms associated with increased Lp(a) levels [111] (Figure 2). A possible explanation is that smaller apo(a) isoforms are secreted more efficiently than the larger ones. However, the relationship between Lp(a) levels and apo(a) isoforms is modified by different SNPs, leading to a great variance in Lp(a) levels of patients with the same apo(a) isoform [119]. Briefly, K-IV_2_ repeat number and SNPs cannot explain Lp(a) levels alone but contribute together to the phenotypic variability.

Recent evidence suggested that high Lp(a)-cholesterol (Lp(a)-c) content is also associated with the presence of a high number of K-IV_2_ repeats [120].

Given that the assessment of K-IV_2_ repeats number remains technically challenging with currently available sequencing technologies, increasing attention has been directed toward SNPs [119]. Among the different SNPs identified through GWAS, rs10455872 and rs3798220 show the strongest association with Lp(a) levels and CVD events. These two SNPs are also associated with smaller apo(a) isoforms. However, in a general Caucasian population of about 3000 individuals it has been observed that 47% of individuals with small apo(a) isoforms do not carry either of these SNPs. Conversely, about 10% of individuals carrying at least one of the two SNPs do not have small apo(a) isoforms. Therefore, approximately half of patients with small apo(a) isoforms would remain undetected if only these two SNPs are genotyped [121,122].

A weighted PRS that aims to predict Lp(a) levels using 43 SNPs in the *LPA* gene has been developed from a GWAS including 48,333 individuals [123]. This Lp(a) PRS was validated in the UK Biobank and demonstrated to be associated with Lp(a) levels in both European and African patients [124]. In a recent study, it has been observed that both the Lp(a) PRS and the LDL-c PRS were higher in hypercholesterolemic patients compared with controls, regardless of the presence of an FH-causative variant [125]. Furthermore, a correlation between the Lp(a) PRS and measured Lp(a) levels has been observed, explaining approximately 45% of their variability [126]. These findings further support the inherited nature of Lp(a) levels. However, SNP characterization and/or the evaluation of apo(a) isoform size (number of repeats) are less efficient and more expensive compared with direct measurement of Lp(a).

In addition, an association between Lp(a) and plaque progression has been reported, independently of LDL-c levels, supporting the role of Lp(a) as a main contributor of the increased CVD risk [121].

### 4.3. Role in Cardiovascular Disease

Different genetic studies, including Mendelian randomization studies, showed that Lp(a) is a causal factor for coronary heart disease (CHD), calcific aortic valve stenosis (CAVS), peripheral arterial disease (PAD), heart failure and all causes of mortality [113].

There is a linear and continuous association between Lp(a) concentration and CVD risk, so there is no formal cut-off that could be used to define high Lp(a) levels. However, 125 nmol/L (50 mg/dL) is often used as a clinically relevant cut-off, as indicated in European and American guidelines [16,101].

It has been observed that patients with Lp(a) levels > 430 nmol/L (>180 mg/dL) have a CVD risk comparable to that of HeFH patients [111]. The relationship between Lp(a) and CVD risk is particularly relevant in the context of FH. In fact, high levels of Lp(a) in FH patients may further increase their lifetime exposure to atherogenic lipoproteins, increasing their residual risk. This could be particularly relevant, considering that the risk per particle has been estimated to be 6–7 times for Lp(a) than for LDL particles [127]. Therefore, considering both quantity and quality of ApoB-containing lipoproteins may improve CVD risk assessment [127].

Beyond its pro-atherogenic properties, Lp(a) has also pro-inflammatory and pro-thrombotic effects that could contribute to the formation and progression of atherosclerotic plaques [128,129]. A recent meta-analysis confirmed the association between elevated Lp(a) levels and increased odds of coronary plaque presence, as well as a significant correlation with accelerated plaque progression. These findings also highlight that increased Lp(a) levels contribute to both plaque burden and plaque vulnerability [130].

#### 4.3.1. Oxidation and Inflammation

Lp(a) atherogenic role is mainly due to its infiltration and accumulation in the arterial wall, like that of the other ApoB-containing lipoproteins. However, its demonstrated major atherogenicity [127] is probably due to other properties, such as oxidation. This could enhance the recognition mediated by macrophage scavenger receptors, promoting foam cell formation and plaque progression [129]. Lp(a) is the major plasmatic carrier of oxidized phospholipids (OxPLs), contributing to its pro-inflammatory role promoting endothelial activation [131]. It has been demonstrated that patients with ASVD and high Lp(a) levels have increased levels of circulating inflammatory mediators compared to patients with low levels of Lp(a), linking monocyte activation to inflammation and thrombosis [129].

#### 4.3.2. Thrombosis

The pro-thrombotic effects of Lp(a) are much debated and are probably due to its homology to plasminogen; in fact, apo(a) contains a protease-like domain like that of plasminogen but inactive. This potential competition with plasminogen could lead to impaired fibrinolysis. However, epidemiological and genetic studies demonstrated that these effects in humans are inconsistent [111].

Supporting this inconsistency, a recent study demonstrated that the association between Lp(a) and MI is not modified by variants in the coagulation either through the platelet or thrombin pathway, highlighting that the increased risk of MI is not due to prothrombotic effects of Lp(a). These findings have important repercussions in clinical practice, in particular in the treatment of patients with high Lp(a) levels, not supporting antiplatelet therapy in primary cardiovascular prevention [132].

### 4.4. Measurement of Lp(a)

Lp(a) levels are usually stable throughout life, so, with some exceptions (kidney or liver diseases, acute infections or physiological conditions like menopause), repeated measures are not required to improve CVD risk prediction [133]. Accordingly, recent European Atherosclerosis Society (EAS) consensus statements and Canadian and American guidelines recommend measuring Lp(a) at least once in adults [16,101,134]. Given its low within-subject variability (10.2%; https://biologicalvariation.eu/, accessed on 7 May 2026), Lp(a) measurement could be intended as a once-in-a-lifetime investigation similarly to genetic testing.

Major differences in Lp(a) levels reported in the literature seem to be caused by analytical issues rather than biological variability [135], making it useful to pay attention to the method used.

#### Analytical Issues Impacting Lp(a) Measurement

Particular attention should be given to the analytical method used for Lp(a) measurement because of the lack of harmonization, i.e., no use of standard measurement units (nmol/L or mg/dL) and no alignment to the reference material used for calibration [136].

Methods and calibration should be set to quantify Lp(a) particle number (mmol/L) and not Lp(a) mass (mg/dL), which is impacted by the size of the apo(a) isoform. Since the conversion between mmol/L and mg/dL depends on the specific apo(a) isoform size, the unit conversion should never be performed.

To improve standardization of Lp(a) measurement, the use of multipoint standards that are traceable to the World Health Organization/International Federation of Clinical Chemistry (WHO/IFCC) reference material, created by a gold standard enzyme-linked immunosorbent assay (ELISA) independent of apo(a) isoform size (SRM 2B) [137], has been recommended [138,139]. Since this serum-derived material is almost depleted, liquid chromatography–mass spectrometry (LC–MS) candidate reference method has been proposed to further standardize analytical methods [140].

Currently, commercially available automated methods based on immunoturbidimetry or immunonephelometry often rely on apo(a) isoform-sensitive assays using polyclonal antibodies that recognize undefined epitopes of apo(a). This could lead to the recognition of the repetitive K-IV_2_ motif of the apo(a), causing an overestimation of large isoforms containing a higher number of repetitions [111].

The ELISA using an antibody against the non-repeated K-IV_9_ for detection is the most reliable for quantification of Lp(a) particles independently from Lp(a) isoform size [141]. It should be noted that this ELISA measures not only apo(a) in Lp(a) but also free apo(a), underestimating the lowering Lp(a) effect obtained by therapeutic molecules that interfere with apo(a) binding to ApoB [142].

To overcome this issue, an isoform-insensitive assay, specific for intact Lp(a) particles, has been developed. This assay is an ELISA that combines antibodies anti-apo(a) and anti-ApoB, allowing the specific detection of intact Lp(a) particles, independently of the number of K-IV_2_ repeats [142]. Unfortunately, this ELISA is not currently commercially available.

It is still not completely clear if only the Lp(a) number should be considered a risk factor or if also its cholesterol content should be considered. The clinical relevance of Lp(a)-c could be its influence on LDL-c measurement, leading to an overestimation of LDL-c levels, especially in patients with high Lp(a) levels [143]. More recently, a direct method to quantify Lp(a)-c has been developed and validated [144,145]. Prior estimation methods assumed that Lp(a)-c is about 30% of Lp(a) mass [146]. A more reliable estimation of Lp(a)-c contribution to LDL-c levels could be obtained using the Rosenson–Marcovina formula [143]. However, this formula also does not account for individual variability of Lp(a)-c and has not been validated across different populations [143]. Further evidence is needed to define the clinical utility of this evaluation.

### 4.5. Lp(a)-Lowering Therapies

Although specific Lp(a)-lowering therapies are still under clinical development, the detection of elevated Lp(a) levels may influence clinical management by supporting a more intensive reduction in residual CVD risk, particularly through optimized LDL-c lowering strategies [101]. In the last decade, different Lp(a) lowering therapies have been developed and are currently under clinical testing. Most of these therapies aim at the inhibition of apo(a) production in the liver using RNA-targeting strategies with single-strand antisense oligonucleotide (ASO) or siRNA [147].

ASOs represent the first class of targeted therapies developed to reduce Lp(a) levels. Pelacarsen is the most used one and acts by inhibiting mRNA production from the *LPA* gene in the hepatocytes, leading to a reduction in Lp(a) levels of about 80%. It has also been demonstrated that this ASO leads to a significant reduction of OxPLs carried by Lp(a), highlighting its potential anti-inflammatory role [131,148].

Another RNA-targeting strategy is the use of siRNA, such as olpasiran and lepodisiran. In the phase 2 trial, it has been observed that olpasiran leads to a reduction in Lp(a) levels of about 95%. A similar reduction has been observed in early-phase trials of lepodisiran [131,149].

A different approach is represented by small molecules such as muvalaplin, which inhibits Lp(a) formation by preventing the interaction between apo(a) and ApoB, leading to a reduction of intact Lp(a) of up to 85.8% [150,151].

All these therapies seem to be very promising in lowering Lp(a) levels. However, further studies are needed to better understand their potential role in the reduction in clinical events preventing cardiovascular diseases.

## 5. Conclusions

The genetic background underlying increased LDL-c levels is complex, being influenced by rare monogenic variants, the cumulative effect of different SNPs with small effects and independent genetic factors like Lp(a). Therefore, a comprehensive evaluation is required to better define genetic predisposition to hypercholesterolemia.

Current evidence has emphasized the importance of integrating clinical data and biochemical characterization with genetic information to improve CVD risk assessment and patients’ management [152].

**Figure 3:**
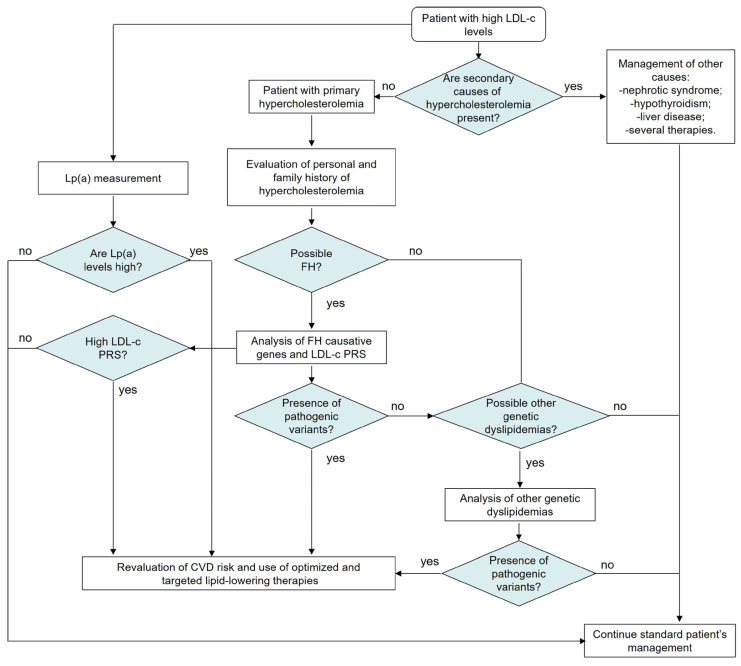
Integrated workflow for the evaluation of patients with high LDL-c levels. In patients with high LDL-c levels, lipoprotein(a)—Lp(a)—measurement should always be performed. After exclusion of secondary causes of hypercholesterolemia, patients should undergo clinical assessment to evaluate the origin of primary hypercholesterolemia. In patients with clinical suspicion of Familial Hypercholesterolemia (FH), genetic analysis of FH-causative genes should be performed, evaluating also the LDL-c polygenic risk score (LDL-c PRS). Analysis of other genetic dyslipidemias may help to explain the phenotype and to define a targeted therapy. The integration of Lp(a) and genetic data could allow the reevaluation of CVD risk and the use of optimized and targeted lipid-lowering therapies.

From a clinical perspective, the evaluation of patients with high LDL-c levels could follow a stepwise approach. Lipoprotein(a)—Lp(a)—measurement should always be considered in all subjects, including hypercholesterolemic ones. After exclusion of secondary causes of hypercholesterolemia (i.e., nephrotic syndrome, hypothyroidism, liver disease, and several therapies [153]), patients should undergo clinical assessment, with evaluation of personal and familial history of hypercholesterolemia, to evaluate the origin of primary hypercholesterolemia. In patients with clinical suspicion of FH, genetic analysis of FH-causative genes should be performed by evaluating also LDL-c PRS that, if high, could be considered both an alternative cause of hypercholesterolemia and a worsening factor of FH genetically confirmed. Evaluation of other genetic dyslipidemias should also be considered to define the most appropriate therapies. The integration of all these data could allow us to improve CVD risk reclassification and to facilitate a more personalized therapeutic approach with the introduction of optimized and targeted lipid-lowering therapies (Figure 3).

## 6. Future Perspectives

AI is emerging as a promising tool in cardiovascular medicine, with increasing applications in data integration and risk prediction [154]. In the context of hypercholesterolemia, AI-based approaches, including machine learning and deep learning models, could facilitate the integration of genetic, biochemical and clinical variables. This could allow us to improve CVD risk stratification, moving an important step towards personalized medicine.

## Figures and Tables

**Table 1:** Monogenic disorders characterized by high LDL-c levels.

Monogenic Hypercholesterolemia	Genes	Inheritance
Familial Hypercholesterolemia (FH)	*LDLR*, *APOB*, *PCSK9*, *APOE*	Autosomal semi-dominant
Autosomal recessive hypercholesterolemia (ARH)	*LDLRAP1*	Autosomal recessive
Sitosterolemia	*ABCG5*, *ABCG8*	Autosomal recessive
Cerebrotendinous xanthomatosis (CTX)	*CYP27A1*	Autosomal recessive
Lysosomal acid lipase deficiency (LALD)	*LIPA*	Autosomal recessive

## Data Availability

No new data were created or analyzed in this study.

## Abbreviations

The following abbreviations are used in this manuscript:

AI	Artificial intelligence
ALT	Alanine aminotransferase
ANGPTL3	Angiopoietin-like protein 3
apo(a)	Apolipoprotein(a)
ApoB	Apolipoprotein B
APOE	Apolipoprotein E
ARH	Autosomal recessive hypercholesterolemia
ARISE	Algorithmic risk inspection for screening elevated Lp(a)
ASCVD	Atherosclerotic cardiovascular disease
ASO	Single-strand antisense oligonucleotide
AST	Aspartate aminotransferase
CAD	Coronary artery disease
CAVS	Calcific aortic valve stenosis
CHD	Coronary heart disease
c-IMT	Carotid intima-media thickness
CTX	Cerebrotendinous xanthomatosis
CVD	Cardiovascular disease
EAS	European Atherosclerosis Society
ELISA	Enzyme-linked immunosorbent assay
ERT	Enzyme replacement therapy
FH	Familial Hypercholesterolemia
GWAS	Genome-wide association study
HDL-c	High-density lipoprotein cholesterol
HeFH	Heterozygous FH
HoFH	Homozygous FH
HSCT	Hematopoietic stem cell transplant
IFCC	International Federation of Clinical Chemistry
K-IV_2_	Kringle IV type 2
LALD	Lysosomal acid lipase deficiency
LC-MS	Liquid chromatography–mass spectrometry
LDL-c	Low-density lipoprotein cholesterol
LDLR	LDL receptor
LDLRAP1	LDLR adaptor protein 1
LLT	Lipid-lowering therapy
Lp(a)	Lipoprotein(a)
Lp(a)-c	Lipoprotein(a) cholesterol
LIPA	Lysosomal acid lipase
MI	Myocardial infarction
MTTP	Microsomal triglyceride transfer protein
NGS	Next Generation Sequencing
Non-HDL-c	Non-High-density lipoprotein cholesterol
NPC1L1	Niemann–Pick C1-like 1
OxPLs	Oxidized phospholipids
PAD	Peripheral arterial disease
PCSK9	Proprotein convertase subtilisin/kexin type 9
PCSK9i	PCSK9 inhibitors
PRS	Polygenic risk score
siRNA	Small interfering RNA
SNPs	Single-nucleotide polymorphisms
USV	Uncertain significance variant
VLDL	Very low-density lipoprotein
WHO	World Health Organization
